# Is There Circulation in the Dentine?

**Published:** 1860-05

**Authors:** E. Collins


### IS THERE CIRCULATION IN THE DENTINE?

BY E. COLLINS.

ASIDE from what we have learned occularly, by the aid of the microscope, respecting a dental circulation, we have other and more infallible criteria by which we are enabled to arrive at that point of definiteness which will justify us in coming to the settled conclusion that the dentine is, in common with other tissues of the human body, endowed with veins and arteries, and that this dense ivory structure is consequently subject to similar changes observable in other tissues.

The criteria, then, to which I desire to call attention, and which I believe fully and satisfactorily to answer this question, are the following:

The dentine, in order to the existence of the vital anatomical relation which it sustains to the rest of the body, must possess, in accordance with the laws of the animal economy, life in itself.

This all are ready to admit, and in so doing, must admit all, or at least the plausibility of what I am about to attempt to prove.

What is life? I answer, it is the same coöperating agencies that gives and sustains animal life. But what, you inquire, are these agencies? I answer, sensation, motion and nutrition. These are dependent for their existence one upon the other, in the living animal tissue, and hence, if there be dentinal nerves—a fact which has been established beyond doubt, there must be necessarily accompanying arteries and veins. For it is not to be reasonably presumed that the nerves of the osseous tissues possess any distinctive characteristics from the nerves of the soft tissues, hence, they must be subject to similar wear and waste of their compound elements, which would soon result in their extinction, was it not that these elements are being constantly supplied, as disintegration takes place, through the medium of a dentinal circulation, the only medium known to us, through which the various elements entering into the formation of the human body are conveyed to their respective destinations. And thus it is that other of the animal elements of dentine exist, and in accordance with first physical laws, is constantly undergoing changes, which take place more or less rapidly, according to the age, temperament and general health of the individual.

Lastly, the fact that the dentine, as age advances, becomes more and more dense, and less sensitive, in consequence of the gradual increase of the earthy and the proportionate decrease of the animal elements, is one with which every careful observer is familiar, and which of itself affords us sufficient proof of the existence of a dentinal circulation.

